# A quasi-experimental test of an intervention to increase the use of thiazide-based treatment regimens for people with hypertension

**DOI:** 10.1186/1748-5908-2-5

**Published:** 2007-02-13

**Authors:** Carol M Ashton, Myrna M Khan, Michael L Johnson, Annette Walder, Elizabeth Stanberry, Rebecca J Beyth, Tracie C Collins, Howard S Gordon, Paul Haidet, Barbara Kimmel, Anna Kolpakchi, Lee B Lu, Aanand D Naik, Laura A Petersen, Hardeep Singh, Nelda P Wray

**Affiliations:** 1 General Medicine Section, Veterans Affairs Medical Center, Houston, Texas, USA; 2 Center for Quality of Care and Utilization Studies, Veterans Affairs Medical Center, Houston, Texas, USA; 3 Section of Health Services Research, Department of Medicine, Baylor College of Medicine, Houston, Texas, USA; 4 Section of General Medicine, Department of Medicine, Baylor College of Medicine, Houston, Texas, USA; 5 Pharmacy, Veterans Affairs Medical Center, Houston, Texas, USA

## Abstract

**Background:**

Despite recent high-quality evidence for their cost-effectiveness, thiazides are underused for controlling hypertension. The goal of this study was to design and test a practice-based intervention aimed at increasing the use of thiazide-based antihypertensive regimens.

**Methods:**

This quasi-experimental study was carried out in general medicine ambulatory practices of a large, academically-affiliated Veterans Affairs hospital. The intervention group consisted of the practitioners (13 staff and 215 trainees), nurses, and patients (3,502) of the teaching practice; non-randomized concurrent controls were the practitioners (31 providers) and patients (18,292) of the non-teaching practices. Design of the implementation intervention was based on Rogers' Diffusion of Innovations model. Over 10.5 months, intervention teams met weekly or biweekly and developed and disseminated informational materials among themselves and to trainees, patients, and administrators. These teams also reviewed summary electronic-medical-record data on thiazide use and blood pressure (BP) goal attainment. Outcome measures were the proportion of hypertensive patients prescribed a thiazide-based regimen, and the proportion of hypertensive patients attaining BP goals regardless of regimen. Thirty-three months of time-series data were available; statistical process control charts, change point analyses, and before-after analyses were used to estimate the intervention's effects.

**Results:**

Baseline use of thiazides and rates of BP control were higher in the intervention group than controls. During the intervention, thiazide use and BP control increased in both groups, but changes occurred earlier in the intervention group, and primary change points were observed only in the intervention group. Overall, the pre-post intervention difference in proportion of patients prescribed thiazides was greater in intervention patients (0.091 vs. 0.058; p = 0.0092), as was the proportion achieving BP goals (0.092 vs. 0.044; p = 0.0005). At the end of the implementation period, 41.4% of intervention patients were prescribed thiazides vs. 30.6% of controls (p < 0.001); 51.6% of intervention patients had achieved BP goals vs. 44.3% of controls (p < 0.001).

**Conclusion:**

This multi-faceted intervention appears to have resulted in modest improvements in thiazide prescribing and BP control. The study also demonstrates the value of electronic medical records for implementation research, how Rogers' model can be used to design and launch an implementation strategy, and how all members of a clinical microsystem can be involved in an implementation effort.

## Background

Hypertension affects half to two-thirds of people older than age 60, and is a major etiologic factor of the leading causes of mortality and morbidity in developed countries [1-3]. Control rates, while improving, are abysmally low [4]. Physicians have a large array of antihypertensive agents from which to choose. These agents vary less in efficacy than they do in cost, a major concern to payers everywhere.

The Antihypertensive and Lipid-Lowering Treatment to Prevent Heart Attack Trial (ALLHAT) was an eight-year, $102 million study funded by the U.S. National Heart, Lung, and Blood Institute (NHLBI) [5]. ALLHAT is the largest randomized trial ever conducted to examine the effects of antihypertensive drugs on clinical outcomes. It included 33,357 participants older than age 55 (47% women, 36% diabetic, 35% black) from 623 North American centers, including 7,067 participants from Veterans Affairs medical centers [6]. ALLHAT showed that thiazide-based antihypertensive regimens are more cost-effective than other regimens, a finding that has been incorporated into current U.S. national hypertension treatment guidelines [7]. The main results of ALLHAT were published on December 18, 2002 to great attention in the medical and lay press [5]. Despite ALLHAT's high-quality evidence, national treatment guidelines incorporating its findings, and extensive press, implementation of ALLHAT findings into routine clinical practice has been insubstantial, judging by the low proportion of hypertensive patients on thiazides (e.g., <13% in a large US health maintenance organization [8]), though some increase in anti-hypertensive diuretic use is occurring [8,9]. On February 1, 2006 the NHLBI announced a three-year, $3.7 million effort to drive ALLHAT results into practice [10], an acknowledgement that ALLHAT's results were having less impact on practice than was hoped.

The U.S. Veterans Health Administration (VHA) is a federally funded comprehensive national health care system that provided services to more than 4.5 million veterans in 2004. About 95% of VA health care users are men, and 51% are older than age 65. The prevalence of chronic conditions is high; for example, 52% of VA beneficiaries in the southeastern U.S. have hypertension and 16% have diabetes [11]. Because of the high prevalence of hypertension in VA beneficiaries and the relatively low rates of thiazide use [12,13], full-scale implementation of thiazide-based antihypertensive regimens as the treatment of choice could significantly improve health outcomes and save considerable costs to our medical center and the VA system. For example, at the VA medical center (VAMC) in Houston, Texas the cost of a 90-day supply of hydrochlorothiazide at 25 mg daily is $0.63, while the cost for a 90-day supply of amlodipine at 2.5 mg daily (one of the drugs tested in ALLHAT) is $63.54.

The goal of our study was to design and test a strategy to implement the results of ALLHAT into routine daily practice, and to add to the emerging body of knowledge on implementation processes, barriers and facilitators. A six-month, $50,000 implementation planning grant from the VA Health Services Research and Development Service (HSR&D) funded the study. A priori, we specified that to be classified as effective, our implementation intervention would have to first, increase the proportion of "eligible" hypertensive patients (those without indications for non-thiazide agents as first-line antihypertensive drugs, specifically those with a serum creatinine <2 mg/dL and no diagnosis of gout or heart failure, the same as specified in ALLHAT), who were prescribed a thiazide-based regimen, and second, increase the proportion of hypertensive patients, regardless of regimen, whose blood pressure was at goal (<140/90; if diabetic, <130/80) at their most recent visit to any clinic at the VA medical center [7,14].

Although thiazides have been recommended for hypertension for decades, we viewed ALLHAT's major finding (that thiazides are the treatment of choice for most patients with hypertension) as an innovation. Therefore, the conceptual framework we used to design our strategy to speed the diffusion of this innovation was Everett Rogers' diffusion model [15]. Rogers' model defines diffusion as the process by which an innovation is communicated through certain channels over time, and posits five attributes of an innovation that drive its rate of adoption: relative advantage, compatibility (e.g., with organizational culture), complexity, trialability (ability to try something out before investing fully), and observability (being able to see the results).

## Methods

### Setting, participants, and data sources

This study was approved by the Institutional Review Board of Baylor College of Medicine and the Research and Development Committee of the VA Medical Center, Houston, Texas. The VAMC in Houston, Texas was the setting for this project. The staff physicians and nurses of the General Medicine Section (GMS) initiated and conducted the study. The GMS staff physicians are board-certified in internal medicine. All provide direct patient care and precept trainees in hospital and clinic settings; one-half also conduct health services research. At the time of the study, the GMS ambulatory practice was a teaching service that included approximately 150 trainee physicians in post-graduate years one to three during any academic year, staff assistants, and roughly 6,000 adult patients. Target panel sizes ranged from 25 patients for interns, 50 for residents, and 100–250 for staff physicians. During the intervention period, which spanned parts of two academic years, 228 unique providers had GMS panels. The GMS and its patients constituted the intervention group. Because the GMS designed the intervention and then instituted it within its own practice, the GMS was the observee, as well as the observer in this study.

The VAMC's non-teaching adult ambulatory general internal medicine service (called "PrimeCare") and its roughly 40,000 patients constituted the concurrent control group. PrimeCare physicians, many of whom are board-certified internists, spend the majority of their time delivering direct care and had no outpatient teaching responsibilities during the study. Target panel sizes ranged from 1000–1600 patients. During the intervention period, PrimeCare had 31 practitioners credentialed as independent providers (24 physicians, 5 physician assistants, and 2 nurse practitioners).

Upon enrollment at the Houston VAMC, an administrator assigned each new patient to a GMS or PrimeCare practitioner's panel on a space-available basis. Each practitioner was responsible for providing assigned patients with continuous and comprehensive primary care. In addition to referring for the usual subspecialty consultations, primary care practitioners could supplement the care of complicated patients by enrolling them in nurse follow-up clinics. Standardized treatment protocols for hypertension were not in use during the study. VA medical records were fully electronic, integrated, and accessible to providers.

The medical center's warehouse of computerized medical record data served as the source of data for identifying patients with the diagnosis of hypertension, excluding those with possible indications for non-thiazide first-line agents (serum creatinine >2 mg/dL, diagnoses of gout and/or heart failure), and for measuring time trends in the two outcome measures. We developed computer algorithms for defining the target population and measuring key variables [11]. We used all patients who met the conditions in our defining algorithms, and did not perform any sample size or power calculations beforehand. The target population consisted of patients who had: a) inpatient or outpatient International Classification of Diseases, Ninth Revision, Clinical Modification codes for hypertension, or b) pharmacy fill records for one or more anti-hypertensive drugs, or, c) in the absence of either of the former, at least two elevated blood pressure measurements. The misclassification rate of these algorithms has not been empirically assessed. The medical center's analyst used our algorithms on the electronic records in the data warehouse. Using these data, our study statisticians conducted all statistical analyses.

### Description of the implementation intervention

The implementation plan was team-based. GMS members were assigned to four teams established to pursue goals derived from the Rogers diffusion model (Table 1). At weekly or biweekly steering committee meetings, each team presented its progress and draft products to obtain feedback and advice. This iterative process ensured that each team benefited from the collective expertise of the GMS and that all GMS members were kept informed. To overcome *complexity*, team developed tangible products (message and vehicles) that would make it easy for a clinician to prescribe thiazides (e.g., pocket cards displaying treatment algorithms for starting or converting to a thiazide). To show *relative advantage *and influence the change, team developed pocket cards showing comparative drug costs, informational posters, lectures, literature repositories about ALLHAT, and electronic reminders in patient charts. To meet needs for *trialability *and *observability*, the group analyzed patterns of prescribing and blood pressure goal attainment in GMS vs. PrimeCare. To expedite *communication *about the innovation, team members served as messengers about ALLHAT with patients, internal medicine trainees, and key medical center entities (Pharmacy and Therapeutics Committee, medical center leadership, director of Pharmacy Service, and physician-directors of non-GMS primary care clinics). We did not explicitly address compatibility, because cost-effective care is a cultural cornerstone in the publicly-funded VA medical care system. The GMS devoted 2,131 person-hours to the study during the 10.5-month intervention period.

**Table 1:** List of project teams and their charges

**Team Name**	**Charge**	**Attribute from Rogers Model**
Steering Committee	Create project timeline and milestones; monitor progress; review and vote on algorithms for starting or switching to thiazides prepared by Clinician Liaison Team; keep project on track; review GMS and PrimeCare data on thiazide use and blood pressure goal attainment	None
Clinician Liaison Team	Using published literature (specifically the JNC7 national guidelines) create algorithms guiding clinicians on how to start or to switch a patient to thiazides. Review and present literature on effectiveness of "academic detailing" to change physician behavior.	Complexity
Patient and Administrator Liaison Team	Provide informational materials to patients and answer their questions; conduct focus groups of patients to determine how they would feel about the use of thiazides, or about changing their antihypertensive regimens to switch to a thiazide. Inform key VA medical center leaders about the project and serve as their point of contact.	Complexity
Communication Team	Using information provided by the Clinician Liaison Team and Steering Committee, format and produce all written materials concerning the project, including treatment-algorithm and drug-cost comparison pocket cards for clinicians, exam room posters and brochures for patients, conference room posters and brochures for clinicians, blood pressure measurement procedure for nurses, and project reports	Relative advantage
Performance Analysis Team	Devise conceptual and measurement models for tracking patient outcomes; and oversee computer programming algorithms to use with the medical center's data warehouse for the prevalence of hypertension, use of thiazides and other anti-hypertensives, and blood pressure goal attainment. Assess and establish data quality. Review and format GMS and PC data on study outcomes.	Observability; trialability

Because most GMS and PrimeCare practitioners worked in adjacent clinic halls and had regular contact, we recognized the probability that some elements of the intervention would "leak" into PrimeCare. During the 10.5-month intervention period, we made no effort to include or to exclude non-GMS practitioners from the intervention, and we shared materials (e.g., pocket cards) whenever asked. After our intervention period ended, we learned that a contemporaneous quality-improvement project aimed at increased thiazide prescriptions was occurring in PrimeCare, consisting of distribution of copies of the ALLHAT main results publication, several lectures on ALLHAT's findings, and the introduction of an electronic reminder to consider a thiazide diuretic into the medical record of every patient whose blood pressure was not controlled at the time of the visit and was not prescribed a diuretic. Thus, the PrimeCare comparison group cannot be considered as a no-treatment control group.

### Analysis periods

We present time series data for thiazide use and blood pressure control in the intervention and control groups from July 2002 through March 2005 (33 months). The active intervention period was November 13, 2003 through September 30, 2004. The July 2002–November 2003 pre-intervention period established the baseline and allowed us to determine whether the December 2002 publication of ALLHAT's main results exerted any effect. The October 2004–March 2005 post-intervention period allowed an examination of sustainability of effects.

### Statistical analysis

We used statistical process control tools [16] to examine trends in thiazide use and BP control. In this analysis the unit of observation was the clinic visit. Because the data were discrete (the proportion of patients prescribed thiazides or achieving BP goals), we used p-type control charts, calculating control limits of +/- 3 standard errors based on the pre-intervention period. In such charts, significant change is indicated when the mean proportion rises (or falls) outside the upper or lower control limits. Using 3 × SE is equivalent to testing using a type 1 error probability alpha = 0.0027, an adequate correction for multiple comparisons.

We also analyzed cumulative sum charts, which can detect subtle changes in time series data missed by control charts, using "Change-Point Analyzer" software [17]. The CUSUM (change point) program uses serial bootstrap sampling of cumulative sums to detect deviations from the range of expected values. When a change is detected, a bootstrap analysis is performed to determine a confidence level for the change and an estimate of when it occurred. A level 1 or primary change represents the first change detected. Any other changes are detected by subsequent passes through the data. We used only level 1 changes to control for multiple testing. The unit of observation for the CUSUM analyses was the clinic visit. Key events that could have inflected the time series lines for thiazide prescribing and BP goal attainment during the observation period are given in Table 2.

**Table 2:** Key events that could have influenced GMS physicians' propensity to use thiazide-based regimens for their hypertensive patients

**Date**	**Event**
December 18, 2002	Main results of ALLHAT is published in JAMA, with extensive coverage of the study in lay press.
Early January 2003	GMS Chief observes a pharmaceutical representative give a pre-clinic talk about amlodipine for hypertension to GMS trainee physicians. Representative's talk is followed by a presentation given by a GMS physician on the ALLHAT trial and the cost-effectiveness of thiazides for hypertension. After discussing this at a section meeting and concluding that trainees are getting mixed messages, GMS decides to ban pharmaceutical representatives from the GMC area.
August 23, 2003	During GM Section meeting, chief announces that a deputy secretary of the Department of Veterans Affairs has stated that he will return any financial saving reaped by increased use of thiazide-based regimens to the VA medical center that generated them. Section discusses opportunity to gain funds to support a much-needed additional clinician. (This financial incentive never materialized.)
October 3, 2003	GMS chief circulates a draft to GMS members describing a potential ALLHAT Implementation Project.
October 9, 2003	During GM Section meeting, section decides to go forward with project as delineated in its October 3 draft. Chief announces possibility that a new "implementation research" funding initiative might be launched by the VA HSR&D in Washington, DC.
October 22, 2003	VA HSR&D issues call for implementation planning-grant proposals, due by November 15, 2003.
November 13, 2003	GMS submits proposal for its ALLHAT Implementation Project to VA HSR&D.
December 17, 2003	GMS notified that it has been awarded a $50,000, six-month grant for its ALLHAT Implementation Project
January 15, 2004	Formal project kickoff meeting with full Steering Committee
June 25, 2004	For the first time group-level data for thiazide use and blood pressure goal attainment in GMS vs. PrimeCare became available for review. Project steering committee (consisting of entire project team) reviews and discusses data. (Panel level data were not available during the intervention period.)
September 30, 2004	End of active implementation efforts.
December 9, 2004	Formal close of ALLHAT Implementation Project at a GM Section meeting.

The third method we used to analyze the data ignored its time series nature, and used the patient as the unit of analysis in a "before and after" analysis. Using z-tests of proportions (2-tailed), we compared data on unique patients seen during the quarter before the intervention started (July-September 2003) with data on unique patients seen a year later, during the final months of the intervention (July-September 2004). Clustering effects are unlikely in GMS panels: the significant number of trainees and their rotation schedule led to a large number of different providers, small numbers of patients per panel, and short exposure of panels to specific physicians. Clustering may have been present in PrimeCare, and we did not adjust the pre-post analyses for it. To the extent such clustering was present, it would have led to a smaller standard error for the pooled proportions, which would have increased the value of the test statistic (z score) and lowered its p value.

In the ALLHAT study, diabetics, older patients, and blacks were less likely to have their BP under control at three years [18]. In our sample the proportion of black patients was roughly the same between General Medicine and PrimeCare, but General Medicine had a significantly higher proportion of diabetics and patients over age 65. Consequently, to adjust for potential confounding factors we performed before-after analyses stratified by the presence or absence of diabetes by age <65 or ≥ 65 years.

## Results

**Figure 1:**
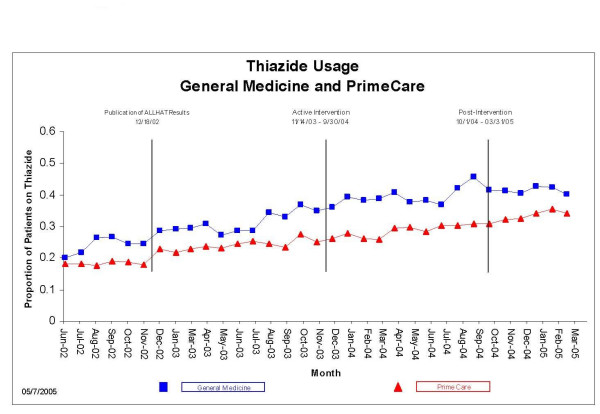
Proportion of hypertensive patients on a thiazide-based regimen, General Medicine vs. PrimeCare, July 2002 through March 2005.

**Figure 2:**
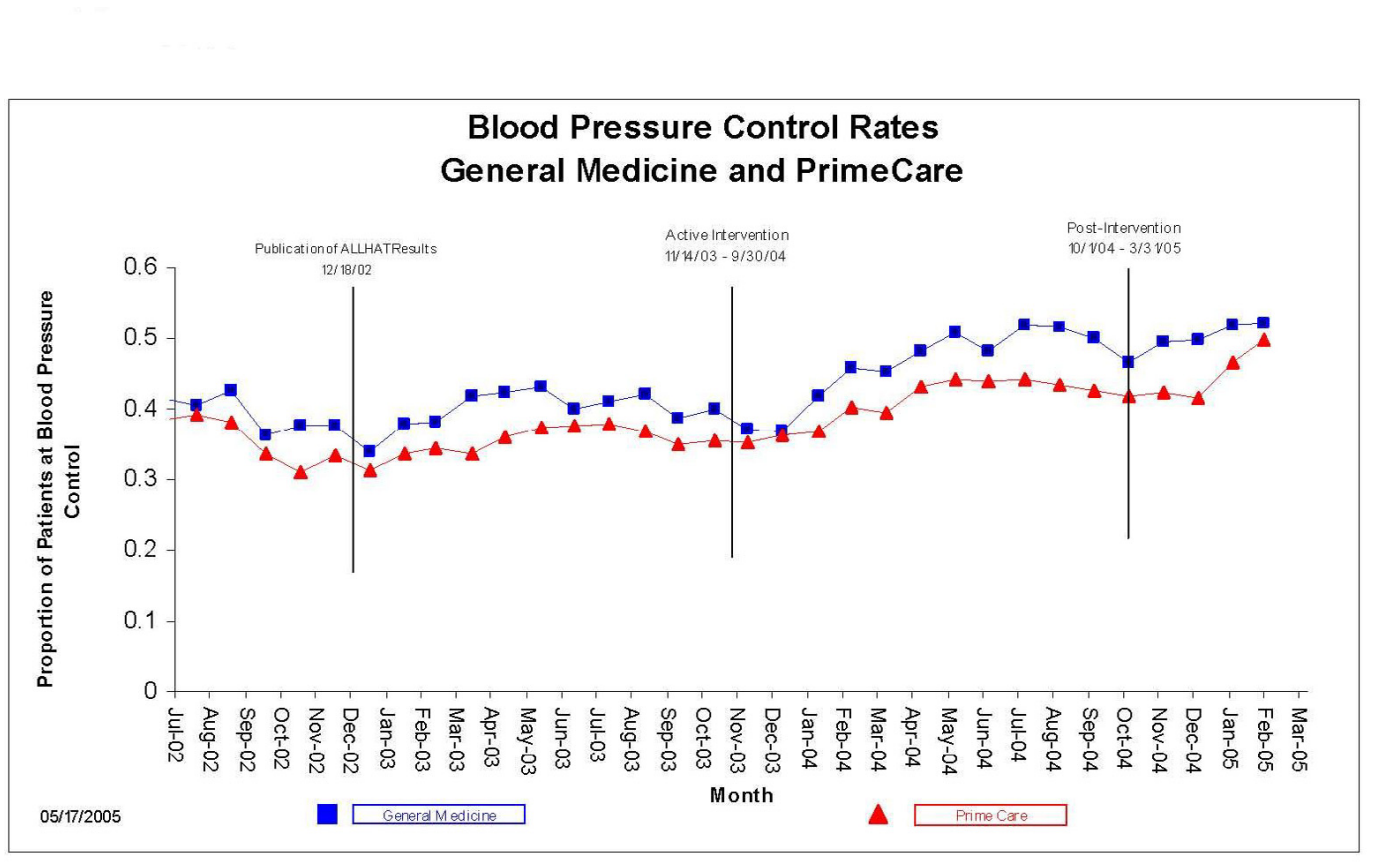
Proportion of hypertensive patients attaining goal blood pressures (<140/90 if nondiabetic; <130/80 if diabetic), General Medicine vs. PrimeCare, July 2002 through March 2005.

Between July 2002 and September 2004, 41,609 unique patients were seen one or more times by GMS and PrimeCare. Of these, 25,047 (60.2%) carried the diagnosis of hypertension; of these, 3,253 were excluded because they were possibly ineligible for thiazides (diagnosed with gout, heart failure, or a serum creatinine >2 mg/dL), leaving 21,794 unique patients. GMS cared for 3,502 (16.1%) and PrimeCare cared for 18,292 (83.9%). GMS patients tended to be older and sicker than PrimeCare patients (Table 3). The proportion of hypertensive patients receiving a thiazide-based regimen, and the proportion of hypertensive patients achieving BP goals were greater in the GMS at baseline and throughout the entire 33-month observation period (Figures 1 &2). In both GMS and PrimeCare patients, the proportion achieving BP goals declined in November-December in all three years, indicating a seasonal effect coinciding with national holidays.

**Table 3:** Characteristics of study patients with hypertension*

	**General Medicine (Intervention)** (n = 3,502)	**PrimeCare (Comparison)** (n = 18,292)	**p**
Male, n (%)	3424 (97.8)	17824 (97.4)	0.2506
			
Race & ethnicity, n (%)			
Non-Hispanic white	2340 (66.8)	12292 (67.2)	<0.0001
Non-Hispanic black	1080 (30.8)	5342 (29.2)	
Hispanic	2 (0.1)	180 (1.0)	
Other or unknown	80 (2.3)	478 (2.6)	
			
Age, mean(SD)	65.3 (11.4)	63.2 (12.2)	<0.0001
			
Age category, n (%)			
<45 years	107 (3.1)	1085 (5.9)	<0.0001
45–64	1563 (44.6)	9173 (50.2)	
65–74	1015 (29.0)	4288 (23.4)	
≥75	817 (23.3)	3746 (20.5)	
			
Coexisting conditions, n (%)			
Diabetes	1796 (40.9)	7039 (33.6)	<0.0001
Ischemic heart disease	1542 (35.1)	4875 (23.3)	<0.0001
Cerebrovascular disease	604 (13.8)	1747 (8.3)	<0.0001
Peripheral arterial disease	520 (11.8)	1714 (8.2)	<0.0001

*Unique patients seen one or more times by General Medicine or PrimeCare practitioners between July 1, 2002 (start of pre-intervention period) and September 30, 2004 (end of intervention period). Numbers will not match those in Table 4 because we studied a dynamic cohort during the 33-month observation period, with patients entering as they met inclusion criteria and exiting due to death or departure from the VA health care system.

### Pre-intervention period: effects on thiazide use of the December 2002 publication of ALLHAT's main results

**Figure 3:**
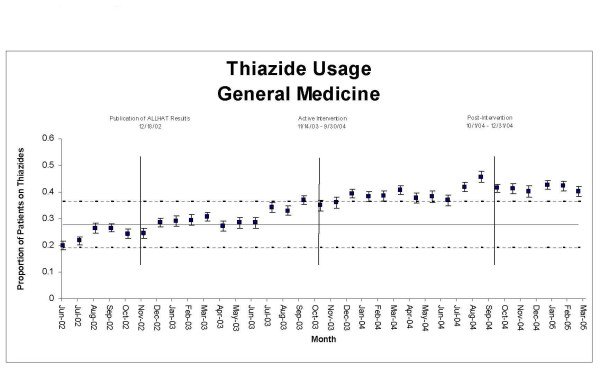
**Statistical process control chart, thiazide usage, General Medicine, July 2002–March 2005**. Upper and lower control limits (dashed lines) are ± 3 SE, based on pre-intervention period.

**Figure 4:**
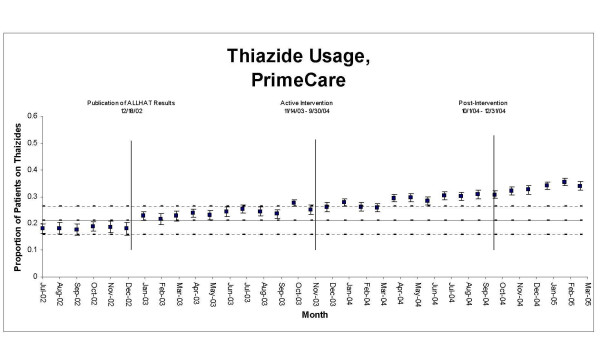
**Statistical process control chart, thiazide usage, PrimeCare, July 2002–March 2005**. Upper and lower control limits (dashed lines) are ± 3 SE, based on pre-intervention period.

Overall, GMS manifested an upward trend in thiazide use throughout the 33 month period; in PrimeCare, thiazide use was flat from July 2002 until December 2002, when it began an upward trend (Figure 1). The increase during the pre-intervention period was not large enough in either GMS or PrimeCare to push the proportion of patients on thiazide-based regimens outside the upper +/-3 SE control limits (Figures 3 &4). However, change-point analysis found a level 1 significant change point with >95% confidence for thiazide prescribing in GMS patients in August 2003, corresponding with the first time an ALLHAT implementation project was discussed at a GM section meeting (Table 2). At this meeting, section members were informed that a deputy VA secretary in Washington had stated that he intended to return any savings achieved by a switch to thiazide-based antihypertensive regimens to the medical center that generated them. Section members discussed this as a potential opportunity to try and generate funds for a much-needed additional clinician. No level 1 significant change points in thiazide prescribing were detected in PrimeCare during the pre-intervention period.

### Intervention period (13 November 2003–30 September 2004): effects of the implementation intervention

During the intervention period, the proportion of GMS and PrimeCare patients on thiazides rose outside the upper limits of their control charts and generally remained there, indicating significant change (Figures 2 &3). The escape from the upper control limits occurred three months earlier in GMS than in PrimeCare. No level 1 change points were detected in thiazide prescribing in GMS or PrimeCare during the intervention period.

**Figure 5:**
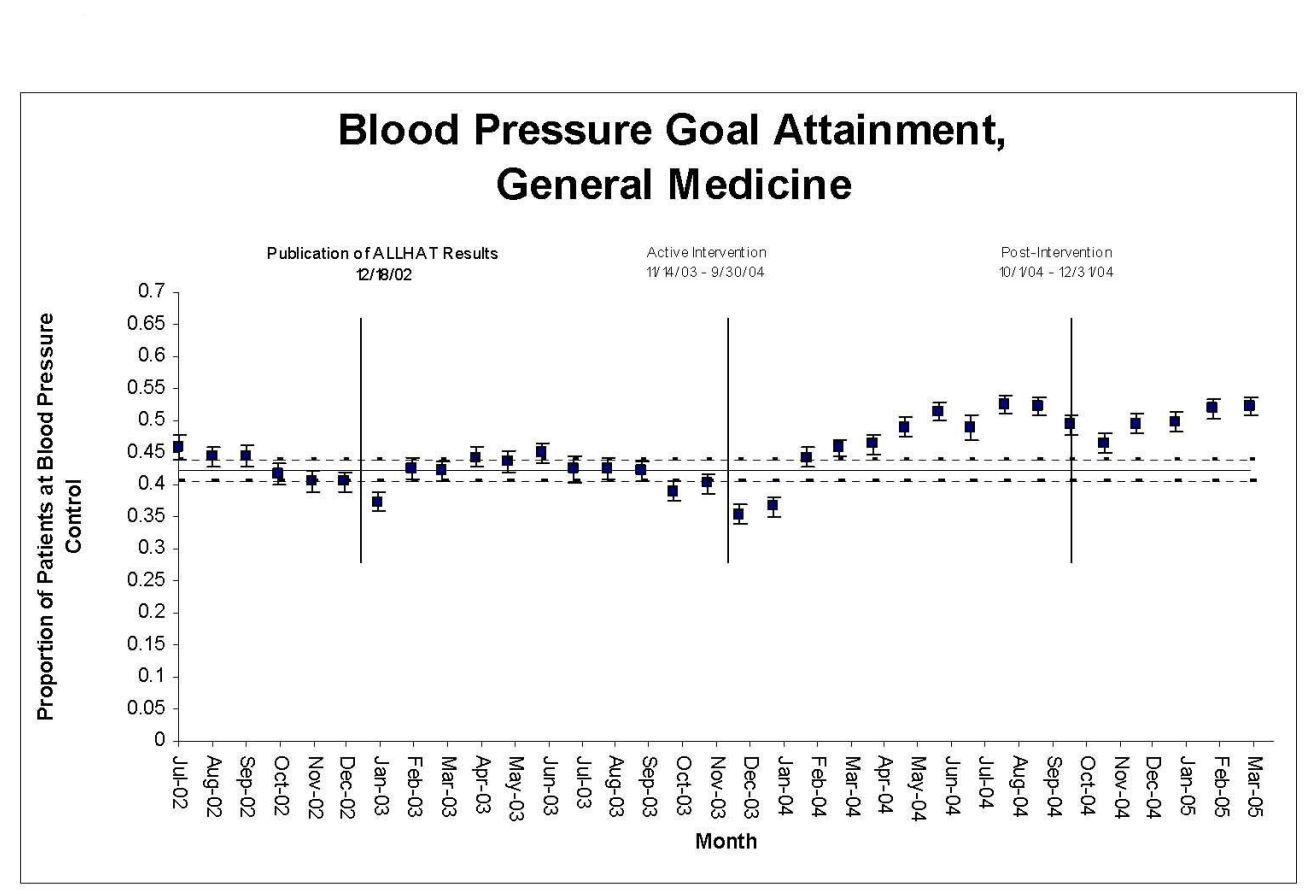
**Statistical process control chart, blood pressure goal attainment, General Medicine, July 2002–March 2005**. Upper and lower control limits (dashed lines) are ± 3 SE, based on pre-intervention period.

**Figure 6:**
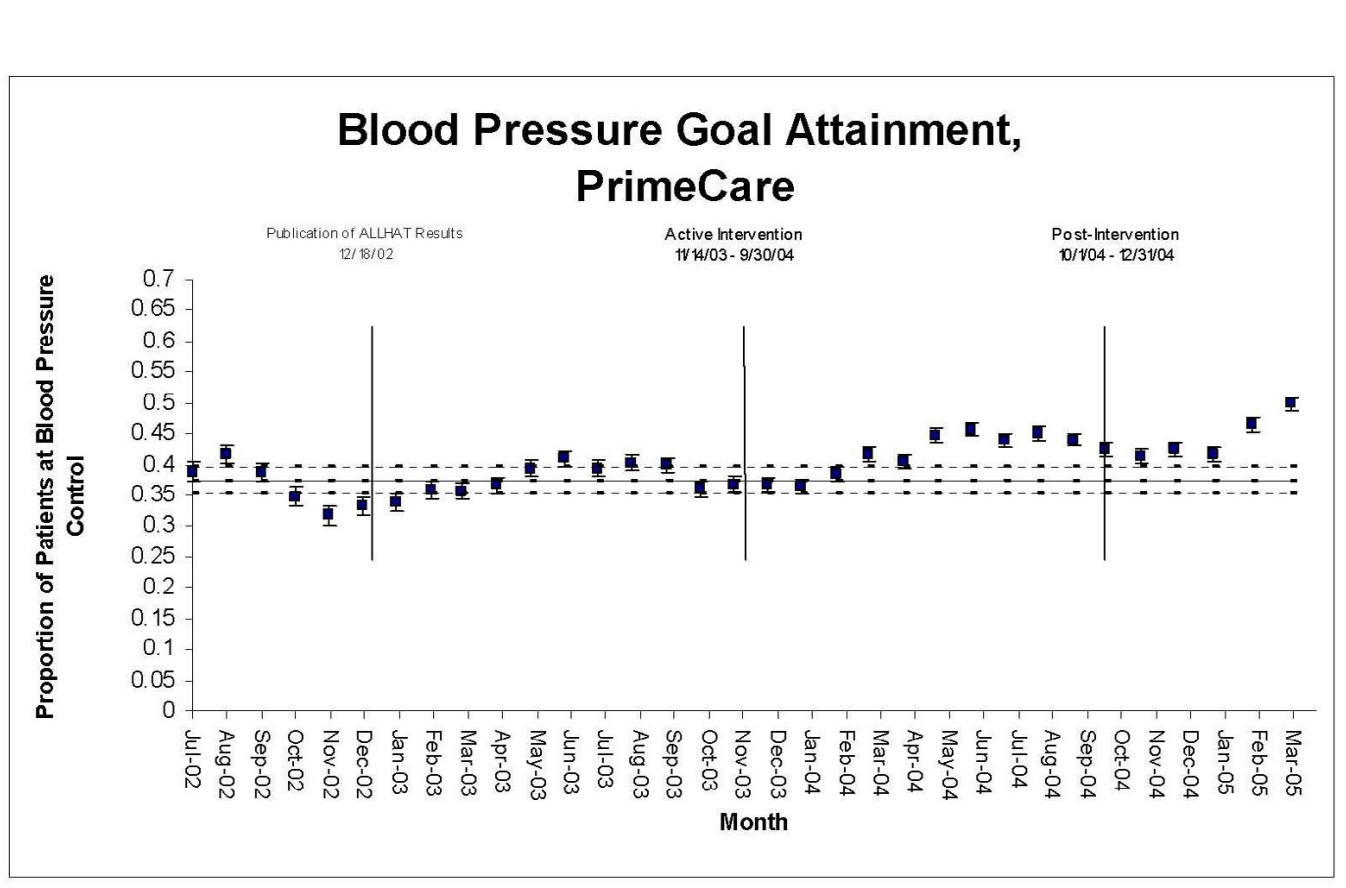
**Statistical process control chart, blood pressure goal attainment, PrimeCare, July 2002–March 2005**. Upper and lower control limits (dashed lines) are ± 3 SE, based on pre-intervention period.

During the intervention period, the proportion of GMS and PrimeCare patients attaining BP goals rose outside the upper limits of their control charts, indicating significant change (Figures 5 &6). The sustained escape from the upper control limits occurred in March 2004 in GMS and in May 2004 in PrimeCare. Change-point analysis detected a level 1 significant change point in GMS patients in March 2004 – a month during which we "saturated" trainees by giving them six pre-clinic lectures on ALLHAT, provided trainees with an ALLHAT reprint collection, and distributed an ALLHAT brochure to all patients. No level 1 change points in BP control rates were detected in PrimeCare.

### Post-intervention period (October 2004–March 2005)

The improvements in thiazide use and in BP goal attainment appear to have been sustained through the end of the observation period (Figures 1 though 6) in GMS as well as PrimeCare.

### Before-after analyses

In a comparison of patients seen in July-September 2004 (toward the end of the intervention period) with those seen during July-September 2003 (before the intervention began), 41.4% (1,232/2,973) of GMS patients were prescribed a thiazide compared with 30.6% (4,154/13,575) of PrimeCare patients (Table 4). The pre- and post-intervention difference in proportion of thiazide use was greater in GMS patients (0.091 vs. 0.058; p = 0.0092). Similarly, a higher proportion of GMS patients achieved BP control compared with PrimeCare: 51.6% (1,535/2,973) versus 44.3% (6,017/13,575), respectively. The pre- and post-intervention difference in BP control was greater in GMS patients (0.092 vs. 0.044; p = 0.0005).

**Table 4:** Before-after analyses* of number and percentages of patients prescribed a thiazide prescribing and patients achieving BP goals regardless of medication regimen, overall and stratified by diabetes status and age older or younger than 65 years.

	**General Medicine (Intervention)**	**PrimeCare (Comparison)**	**p**
**PRESCRIBED A THIAZIDE**, n(%)			
*All Patients*			
Before intervention	845/2616 (32.3)	3103/12510 (24.8)	
After intervention	1232/2973 (41.4)	4154/13575 (30.6)	
Pre-post difference in proportions	0.091	0.058	0.0092
			
*Among Diabetics < 65*			
Before intervention	159/464 (34.3)	523/2209 (23.7)	
After intervention	203/500 (40.6)	794/2581 (30.8)	
Pre-post difference in proportions	0.063	0.071	0.5939
			
*Among Diabetics ≥ 65*			
Before intervention	209/543 (38.5)	522/1897 (27.5)	
After intervention	260/600 (43.3)	581/1877 (31.0)	
Pre-post difference in proportions	0.048	0.035	0.3451
			
*Among Non-Diabetics < 65*			
Before intervention	203/687 (29.5)	1041/4449 (23.4)	
After intervention	351/888 (39.5)	1611/5405 (29.8)	
Pre-post difference in proportions	0.100	0.064	0.0790
			
*Among Non-Diabetics ≥ 65*			
Before intervention	274/922 (29.7)	1017/3955 (25.7)	
After intervention	418/985 (42.4)	1168/3712 (31.5)	
Pre-post difference in proportions	0.127	0.058	0.0021
			
**AT BLOOD PRESSURE CONTROL, n(%)**			
*All Patients*			
Before intervention	1108/2616 (42.4)	4992/12510 (39.9)	
After intervention	1535/2973 (51.6)	6017/13575 (44.3)	
Pre-post difference in proportions	0.092	0.044	0.0005
			
*Among Diabetics < 65*			
Before intervention	141/464 (30.4)	537/2209 (24.3)	
After intervention	197/500 (39.4)	738/2581 (28.6)	
Pre-post difference in proportions	0.090	0.043	0.0778
			
*Among Diabetics ≥ 65*			
Before intervention	128/543 (23.6)	435/1897 (22.9)	
After intervention	224/600 (37.3)	496/1877 (26.4)	
Pre-post difference in proportions	0.137	0.035	0.0004
			
*Among Non-Diabetics < 65*			
Before intervention	341/687 (49.6)	2146/4449 (48.2)	
After intervention	531/888 (59.8)	2846/5405 (52.6)	
Pre-post difference in proportions	0.102	0.044	0.0265
			
*Among Non-Diabetics ≥ 65*			
Before intervention	498/922 (54.0)	1874/3955 (47.4)	
After intervention	583/985 (59.2)	1937/3712 (52.2)	
Pre-post difference in proportions	0.052	0.048	0.4374

* The active intervention began 14 November 2003 and ended 30 September 2004. "Before intervention" denotes unique patients seen during the 90-day period from 1 July through 30 September 2003. "After intervention" denotes unique patients seen during the final 90 days of the active intervention, from 1 July through 30 September 2004.

The stratified analyses (Table 4) show that in both GMS and PrimeCare more diabetics than nondiabetics were prescribed a thiazide at baseline. Proportions increased modestly after the intervention, but the magnitude of the pre- and post-intervention increases in diabetics patients did not differ significantly between GMS and PrimeCare.

Though diabetics were more likely to be prescribed thiazides, they were less likely than non-diabetics to have their BP under control at baseline, as well as after the intervention (Table 4). Among diabetics younger than age 65, the percentage of patients at BP control in GMS increased after the intervention from 30.4% to 39.4%, and in PrimeCare from 24.3% to 28.6%; the pre- and post-intervention differences in proportions (0.090 and 0.043, respectively) did not achieve statistical significance (p = 0.0778). Among diabetics *older than *age 65, the percentage of patients at BP control in GMS increased after the intervention from 23.6% to 37.3%, and in PrimeCare from 22.9% to 26.4%; the pre- and post-intervention differences in proportions (0.137 and 0.035, respectively) achieved statistical significance (p = 0.0004).

## Discussion

Though the effects of the intervention were small, we conclude that our multi-faceted intervention did increase thiazide prescribing and BP control rates in GMS. In this quasi-experimental study, we used three methods (statistical control charts, change-point analysis, and pre-post analyses) to estimate the effects of the intervention on thiazide prescribing and BP control rates in the intervention group. Thiazide prescribing and BP control rates moved out of the upper limits of their control charts several months earlier in GMS than in PrimeCare. Change-point analyses detected two first-level deviations in GMS (one in thiazide prescribing and the other in BP control rates) but none in PrimeCare. Pre-post analyses indicated that the proportion of patients prescribed thiazides increased by 0.091 in GMS compared with 0.058 in PrimeCare, while the proportions of patients achieving BP control increased by 0.092 in GMS and 0.044 in PrimeCare.

The 9.1% increase in persons prescribed thiazides is comparable to what was found in a two-by-two factorial randomized controlled trial in 200 family practices in British Columbia [19]. In that study, thiazide prescribing increased by 11.5% in the practices assigned to receive personalized feedback on prescribing patterns plus an educational module. At the end of our intervention period, more than 40% of GMS patients were prescribed a thiazide, a much higher proportion than the 10–12% reported from another large US health care system [8].

Regarding changes in BP goal achievement rates, if we take the increase in PrimeCare as an indicator of the upward secular trend, an improvement of 4.4% in BP goal attainment rates would have been expected without our intervention. The intervention group manifested a 9.2% increase, thus we conclude that our intervention was associated with a modest increase in BP goal attainment rates. Because relatively small reductions in blood pressure translate into meaningful reductions in the risk of stroke, heart attack, and heart failure, the results of our intervention, if sustained, will be clinically significant for our patients [7]. Many interventions aimed at improving blood pressure control in hypertension have been tested [20-23]. Their results are variable, but many have found larger effects than our study. Nevertheless, the 51.6% of intervention patients who achieved goal blood pressures in this project approaches the 55.2% achieved at one year in the ALLHAT [18] – a randomized trial that used a less stringent goal for diabetics with hypertension. Moreover, it is substantially better than national survey data for control at the 140/90 mmHg threshold: 29% of patients in the U.S., 17% in Canada, and ≤ 10% in Europe [4].

Because this study was performed in a VA setting, almost all patients were men; however, the proportions of black patients (29.5%) and patients with diabetes (40.5%) were roughly similar to ALLHAT's (35% black, 36% diabetic). Another aspect of this study that deserves note is that it was performed in a tax-supported public health care setting in which health care cost-containment is explicitly valued and frequently discussed. Our findings are probably not generalizable to fee-for-service practice settings.

The effect of our intervention on thiazide use and BP goal attainment was probably muted because of the improvements that were occurring in PrimeCare due to their own quality enhancement projects for thiazide use and hypertension control and, possibly, to contamination from our project. This points out the importance of not assuming that practice patterns in a real-world "usual care" comparison groups will be in a steady state – and nor should they be in a culture of continuous quality improvement. In addition, the GMS group had a higher percentage of patients with diabetes (40.9%) compared with PrimeCare (33.6%), and BP goals for diabetics are more stringent (130/80). As the stratified analyses show, the implementation intervention in GMS led to real progress in BP goal achievement in persons with diabetes. Our experience attests to the challenges inherent in real-world implementation projects aimed at improving quality of care, and the importance of including a comparison group when testing a quality improvement intervention.

Implementation studies must often be conducted under conditions in which randomized designs are difficult or impossible to use and contamination is impossible to prevent. Ours was a non-randomized study, and bias may have affected the results. The fact that GMS patients were older and more likely to have diabetes than PrimeCare patients would have made it harder for GMS doctors and patients to increase thiazide use and achieve BP goals over the observation period. However, features of practitioners also were different between the groups, and the extent to which differences in their behavior explain the consistently higher proportion of GMS patients on thiazides and meeting blood pressure goals cannot be determined from this study design. Another limitation of this study is that we could not assess the influence of any individual element of our intervention. To the extent that different elements of a multi-faceted intervention can be introduced in sequence, time series data such as we used might help researchers discern which elements were most powerful and thereby worthy of retention. This small study focused on designing a multi-faceted intervention and putting it to its initial test. In fact, it is important to note that we developed the intervention during the study rather than before we began. In a real sense, developing the intervention was the intervention itself. Testing the influence of any particular aspect of the intervention will require a larger study with a stronger design.

## Conclusion

Our study adds to the empirical and theoretical base of implementation science in several ways. First, it demonstrates the enormous value of electronic medical record systems for interventions aimed at improving practice. Electronic medical records made it possible for us to study nearly 22,000 unique patients with a modest grant of $50,000. Second, it shows the value and the limitations of Rogers' model for the diffusion of innovations, when that model is used in a clinical setting. Because physicians must prescribe thiazides and patients must agree to use them, our intervention focused on communicating the ALLHAT's major findings to them, and ensuring they found it acceptable and easy to use them in practice. The Rogers model proved helpful in structuring the elements, processes, and products of our intervention. However, it was not helpful when we faced trying to "institutionalize" the elements of our implementation intervention into the daily GMS practice without the benefit of external research funding. At that point our efforts should have been guided by a model of organizational change rather than a model for the diffusion of innovations. Using Rogers' model to guide the development and launch of the implementation plan and then shifting to a model of organizational change to guide activities directed at sustaining beneficial change would have been a more effective approach. Third, our study illustrates how all front-line members of a practice – patients, doctors, nurses, and other professionals – can be engaged and can contribute meaningfully in efforts to implement high-quality research evidence into clinical practice, corroborating the Institute of Medicine's assertion that practice improvement happens within such microcosms of care, or happens not at all [24].

## Competing interests

Michael Johnson and Nelda Wray received grant funding for an unrelated study from Pfizer, Inc.; neither feel that this influenced them in the conduct of our ALLHAT implementation study. None of the other authors has a competing financial interest that could have influenced their interpretation of the results of this study.

## Authors' contributions

CMA designed the project, obtained funding, led the project team, and drafted the manuscript. MJK and MLJ planned and conducted all statistical analyses, and AW did the statistical programming. All the authors served as members or leaders of one or more of the implementation teams, helping to design and produce the elements of the implementation intervention and bring those elements into use. All the authors worked together to coordinate the project as members of the Steering Committee. All the authors reviewed the data and helped to interpret the findings. All authors read and approved the final manuscript.
